# Neuropeptide S (NPS) neurons: Parabrachial identity and novel distributions

**DOI:** 10.1002/cne.25400

**Published:** 2022-08-29

**Authors:** Dake Huang, Richie Zhang, Silvia Gasparini, Miriam C. McDonough, William J. Paradee, Joel C. Geerling

**Affiliations:** ^1^ Department of Neurology University of Iowa Iowa City Iowa; ^2^ Department of Neuroscience and Pharmacology University of Iowa Iowa City Iowa; ^3^ Genome Editing Core Facility University of Iowa Iowa City Iowa

## Abstract

Neuropeptide S (NPS) increases wakefulness. A small number of neurons in the brainstem express *Nps*. These neurons are located in or near the parabrachial nucleus (PB), but we know very little about their ontogeny, connectivity, and function. To identify *Nps*‐expressing neurons within the molecular framework of the PB region, we used in situ hybridization, immunofluorescence, and Cre‐reporter labeling in mice. The primary concentration of *Nps*‐expressing neurons borders the lateral lemniscus at far‐rostral levels of the lateral PB. Caudal to this main cluster, *Nps*‐expressing neurons scatter through the PB and form a secondary concentration medial to the locus coeruleus (LC). Most *Nps*‐expressing neurons in the PB region are *Atoh1*‐derived, *Foxp2*‐expressing, and mutually exclusive with neurons expressing *Calca* or *Lmx1b*. Among *Foxp2*‐expressing PB neurons, those expressing *Nps* are distinct from intermingled subsets expressing *Cck* or *Pdyn*. Examining *Nps* Cre‐reporter expression throughout the brain identified novel populations of neurons in the nucleus incertus, anterior hypothalamus, and lateral habenula. This information will help focus experimental questions about the connectivity and function of NPS neurons.

## Graphical abstract

Neuropeptide S (NPS) neurons in the parabrachial region are a subset of *Atoh1*‐derived, *Foxp2*‐expressing neurons that do not express *Pdyn*, *Calca*, or *Cck*. We also identified novel populations of NPS neurons from Cre‐reporter expression in the nucleus incertus, anterior hypothalamus, and lateral habenula.

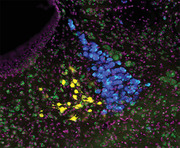

## 1 INTRODUCTION

Wake‐promoting stimulants like caffeine and amphetamines are inherently anxiogenic. Despite the benefits of enhanced wakefulness and attention, their anxiogenic propensity limits therapeutic potential in patients with inattention or hypersomnolence. Conversely, sedative effects of most anxiolytic drugs limit their use in patients with insomnia or anxiety. These complementary limitations frustrate the treatment of many patients with cognitive, affective, and sleep disorders. A nonanxiogenic stimulant medication or nonsedative anxiolytic medication would be enormously beneficial. For these reasons, the discovery of neuropeptide S (NPS) was an exciting development. NPS was identified as an endogenous ligand for the orphan G‐protein‐coupled receptor GPR154 (now NPSR1) and found to both increase wakefulness and reduce anxiety‐related behaviors in mice (Xu et al., 2004).

Human *NPSR1* polymorphisms have been linked to panic disorder and anxiety, as well as asthma, endometriosis, and other inflammatory disorders (Domschke et al., 2011; Donner et al., 2010; Gottlieb et al., 2007; Laitinen et al., 2004; Okamura et al., 2007; Tapmeier et al., 2021). Homozygosity for an *NPSR1* variant that increases sensitivity to NPS (N107I; Reinscheid et al., 2005) was associated with a later bedtime (Gottlieb et al., 2007) and a 20‐min reduction in sleep duration (Spada et al., 2014). Subsequent investigators identified a separate, rare mutation shared by a father and son who were short‐sleepers (4−5 h per night). Replacing a single copy of mouse *Npsr1* with this human variant (Y206H) enhanced responsivity to NPS, increased wakefulness by more than an hour per day, and prevented a memory deficit that normally occurs after sleep deprivation (Xing et al., 2019).

Relative to this genetic and pharmacologic information about its receptor, we know little about the neurons that produce NPS. In rodents and humans, NPS neurons were found in a similar brainstem region (Adori et al., 2015; Clark et al., 2011). The initial report (Xu et al., 2004) used in situ hybridization to identify *Nps*‐expressing neurons near the locus coeruleus (LC) in rats. *Nps*‐expressing neurons lacked gene expression that identifies catecholaminergic neurons in the LC, and likely release the fast‐excitatory neurotransmitter glutamate because they coexpress a vesicular glutamate transporter, not the enzymes that synthesize GABA (Xu et al., 2007). These reports in rats and a study in mice (Clark et al., 2011) also identified a rostral cluster of *Nps*‐expressing neurons. Rostral NPS neurons reportedly occupy the parabrachial nucleus (PB) and principal sensory trigeminal nucleus in rats (Xu et al., 2004; 2007) and the Kölliker‐Fuse nucleus in mice (Clark et al., 2011; Liu et al., 2011). It is unclear whether these neuroanatomical discrepancies reflect species differences or interpretational differences within this complex region of the brainstem.

Currently, we lack the neuroanatomical and genetic information necessary to identify NPS neurons within the molecular framework of the PB region. Such information would improve our understanding of NPS neurons, the region they inhabit, and the neural circuits connected to this region. This information would be useful for designing and interpreting genetically targeted experiments that distinguish the connectivity and function of NPS neurons from other neurons in this complex and diverse region.

The PB region contains two macropopulations of glutamatergic neurons, which derive from separate embryonic precursors (Karthik et al., 2022). Adult neurons generated from these precursors form intermingled yet mutually exclusive populations. Each macropopulation contains subpopulations distinguished by expression of neuropeptides, receptors, and other genetic markers (Garfield et al., 2014; Geerling et al., 2016; Grady et al., 2020; Huang et al., 2020; 2021; Karthik et al., 2022; Miller et al., 2012; Palmiter, 2018). Many other neuronal populations closely surround the PB. These include the LC, laterodorsal tegmental nucleus, mesencephalic trigeminal nucleus, and Barrington's nucleus, but it remains unclear how NPS neurons relate to these populations.

Based on their location and distribution in the PB region, we hypothesized that *Nps*‐expressing neurons are a subset of *Atoh1*‐derived neurons. To test this hypothesis and better characterize NPS neurons, we used a combination of in situ hybridization, immunolabeling, and Cre fate‐mapping for *Atoh1*. To enable genetically targeted experiments involving these neurons, we also generated mice with Cre recombinase knocked into the endogenous *Nps* locus and crossed these mice to a Cre‐reporter strain, which allowed us to identify cells with a history of *Nps* expression.

## 2 MATERIALS AND METHODS

### 2.1 Mice

For neuroanatomical analysis, we used 9 C57BL/6J mice (8−12 weeks, male, Jackson Laboratories), 2 Atoh1‐Cre;R26‐LSL‐L10GFP mice (7 weeks, male and female; Fritzsch et al., 2005; Krashes et al., 2014), and 10 heterozygous Nps‐2A‐Cre;R26‐lsl‐L10GFP mice (6−15 weeks). For additional information about Cre‐driver and ‐reporter mice, see Table 1. All mice were group‐housed in a temperature‐ and humidity‐controlled room on a 12/12‐h light/dark cycle with ad libitum access to water and standard rodent chow (Envigo 7013). All experiments were conducted in accordance with the guidelines of the Institutional Animal Care and Use Committees at the University of Iowa.

**TABLE 1:** Cre‐driver and ‐reporter mice

Strain	References	Source information	Key gene
Atoh1‐Cre	Fritzsch et al. (2005)	Bernd Fritzsch, University of Iowa; Jax 011104	Transgenic construct containing the JQ2‐*Atoh1* promoter fragment ligated to the bacteriophage P1 Cre recombinase
R26‐LSL‐L10GFP Reporter	Krashes et al. (2014)	Available from originating investigators http://www.informatics.jax.org/allele/MGI:5559562	Floxed transcription STOP cassette followed by EGFP/Rpl10 fusion reporter gene under control of the CAG promoter targeted to the Gt(ROSA)26Sor locus
Nps‐2A‐Cre	Generated in this study	Geerling Lab, University of Iowa	2A‐Cre inserted downstream of the endogenous neuropeptide S gene

### 2.2 Generation of Nps‐2A‐Cre mice

**FIGURE 1:**
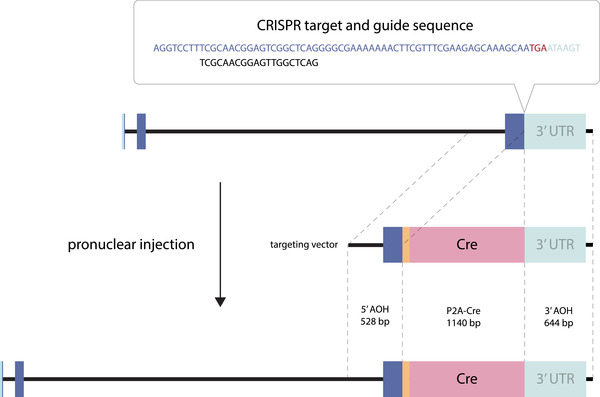
Gene targeting strategy for generation of Nps‐2A‐Cre knockin mice. See Section 2 for details. The three *Nps* exons are blue, 5′ and 3′ untranslated regions (UTR) are light teal, and P2A is orange. Other abbreviations: AOH, arm of homology; bp, base pairs

Knockin Nps‐2A‐Cre mice were generated (Figure 1) in the University of Iowa Genome Editing Core Facility, using the *Easi‐*CRISPR method (Miura et al., 2018). C57BL/6J mice were purchased from The Jackson Laboratory (000664; Bar Harbor, ME). Male mice older than 8 weeks were bred with 3−5‐week‐old superovulated females to produce zygotes for pronuclear injection. Female ICR [Envigo; Hsc:ICR(CD‐1)] mice were used as recipients for embryo transfer.

Chemically modified CRISPR‐Cas9 crRNAs and CRISPR‐Cas9 tracrRNA were purchased from IDT [Alt‐R® CRISPR‐Cas9 crRNA; Alt‐R® CRISPR‐Cas9 tracrRNA (Cat# 1072532)]. The crRNAs and tracrRNA were suspended in T10E0.1 and combined to 1 μg/μl (∼29.5 μM) final concentration in a 1:2 (μg:μg) ratio. The RNAs were heated at 98°C for 2 min and allowed to cool slowly to 20°C in a thermal cycler. The annealed cr:tracrRNAs were aliquoted to single‐use tubes and stored at −80°C.

Cas9 nuclease was also purchased from IDT (Alt‐R® S.p. HiFi Cas9 Nuclease). Cr:tracr:Cas9 ribonucleoprotein complexes were made by combining Cas9 protein and cr:tracrRNA in T10E0.1(final concentrations: 300 ng/μl (∼1.9 μM) Cas9 protein and 200 ng/μl (∼5.9 μM) cr:tracrRNA). The Cas9 protein and annealed RNAs were incubated at 37°C for 10 min. The RNP complexes were combined with single‐stranded repair template and incubated an additional 5 min at 37°C. The concentrations in the injection mix were 30 ng/μl (∼0.2 μM) Cas9 protein, 20 ng/μl (∼0.6 μM) cr:tracrRNA and 20 ng/μl single stranded repair template. Silent mutations were introduced in the protospacer adjacent motif (PAM) sequence and remaining *Nps* codons to reduce homology between the target and wild‐type sequence (Miura et al., 2018).

Pronuclear‐stage embryos were collected using methods described in Pinkert (2002). Embryos were collected in KSOM media (Millipore; MR101D) and washed three times to remove cumulus cells. Cas9 RNPs and single‐stranded repair template were injected into the pronuclei of the collected zygotes and incubated in KSOM with amino acids at 37°C under 5% CO_2_ until all zygotes were injected. A total of 15−25 embryos were immediately implanted into the oviducts of pseudo‐pregnant ICR females.

Founders were identified using Phusion high fidelity DNA polymerase (NEB; M0530) with PCR primers Nps_901 (CTTTAGTACGTGCCACTCTATCC) and Cre_1002 (TCATCCTTAGCGCCGTAAATC) to amplify across the 5′ arm of homology and primers Nps_902 (CGGGCAGTATTTCATGGTTTAAG) and Cre_1001 (CTGACGGTGGGAGAATGTTAAT) to amplify across the 3′ arm of homology. PCR products were cloned into pCR‐Blunt‐II using the Zero Blunt TOPO PCR cloning kit from Thermo Fisher (Cat. K282020). Four to six clones were Sanger‐sequenced to confirm the integrity of the targeted allele. Sequence‐confirmed founders were back‐crossed to wild‐type C57BL/6J mice, and the targeted allele was resequenced in the N1 generation.

### 2.3 Perfusion and tissue sections

All mice were deeply anesthetized with ketamine‐xylazine (i.p. 150‐15 mg/kg) then perfused transcardially with phosphate‐buffered saline (PBS, prepared from 10× stock; P7059, Sigma), followed by 10% formalin‐PBS (SF100, Fisher Scientific). All perfusions for this study were performed during the light cycle, between ZT6−12. After perfusion, the brain was removed and fixed in 10% formalin‐PBS at 4°C overnight, then submerged in 30% sucrose‐PBS at 4°C for an additional day. Each brain was sectioned into 40 μm‐thick axial (coronal) slices using a freezing microtome. Three adjacent (1‐in‐3) tissue series were collected from each brain in separate tubes containing a cryoprotectant solution of 35% (v/v in PBS) ethylene glycol (102466, Sigma‐Aldrich) and 25% glycerol (G22025, RPI). These tubes were stored at −20°C until further processing.

### 2.4 Fluorescence in situ hybridization

To label various mRNA transcripts, we used RNAscope probes listed in Table 2 with the Fluorescent Multiplex Detection Reagents kit (ref# 320851; Advanced Cell Diagnostics). The afternoon before hybridization, we removed one series of tissue sections from cryoprotectant and selected 6−8 sections through the PB region, ranging from a midbrain level immediately caudal to the decussation of the superior cerebellar peduncle back to the level where this white matter tract emerges from the cerebellum. For sections from Nps‐2ACre;R26‐lsl‐L10GFP mice, we choose 2 sections containing the paraventricular hypothalamic nucleus and lateral habenular nucleus, 2 sections from the rostral PB, and 2 sections from the caudal PB. Before mounting and drying sections, sections were wet‐mounted and inspected under an epifluorescence microscope to identify sections containing the most GFP‐expressing neurons in each region.

**TABLE 2:** RNAscope probes used for fluorescence in situ hybridization

Probe	Common name	Channel	ACD catalog #	Lot #
Mm‐Calca‐C3	Calcitonin gene‐related peptide	C2	417691‐C2	18165A
Mm‐Cck‐C2	Cholecystokinin	C2	318771‐C2	16347A
Mm‐FoxP2‐C3	Forkhead Box Protein 2	C3	428791‐C3	17013A
Mm‐Nmb‐C1	Neuromedin B	C1	459931	17060A
Mm‐Nps‐C1	Neuropeptide S	C1	485201	16291A
Mm‐Nps‐C2	Neuropeptide S	C2	485201‐C2	17290
Mm‐Pdyn‐C1	Prodynorphin	C1	318771	19084B
Mm‐Ubc‐C3	Ubiquitin‐C	C3	310771‐C2	Variable

After washing sections briefly in PBS at room temperature (RT), we mounted them on glass slides to dry overnight. In the morning, we outlined sections using a Super‐HI PAP pen (Research Products Incorporated) to form a hydrophobic barrier, and then we washed sections for 2 min twice in PBS at room temperature. The sections were then covered with Protease IV and placed in a covered glass Petri dish, floating in a 40°C water bath, for 30 min. After washing twice in PBS, the sections were then incubated in a combination of 2 or 3 probes for 2 h at 40°C. After that, AMPs 1−4 were added, in series, for 15−30 min each, at 40°C, with two, 2‐min rinses in 1× RNAscope Wash Buffer (#320058; diluted 1:50 in ddH20) between each step. After a final wash in PBS, the slides were dried at room temperature and coverslipped using Vectashield with DAPI. For sections from Nps‐2A‐Cre;R26‐lsl‐L10GFP mice, we performed immunofluorescence labeling for L10GFP before we coverslipped the slides. These slides were washed twice with PBS for 2 min then incubated in a primary antibody solution containing chicken anti‐GFP (Table 3) overnight at 4°C. After washing twice with PBS the following morning, we added Alexa488‐conjugated donkey anti‐chicken IgG (1:500, Jackson ImmunoResearch, Cat# 703‐545‐155, RRID:AB_2340375) for 2 h at room temperature, then washed twice in PBS. After the final PBS wash, we coverslipped the slides using Vectashield with DAPI.

**TABLE 3:** Antisera

Antigen	Immunogen description	Source, host species, RRID	Concentration
Choline acetyltransferase	Human placental choline acetyltransferase	Millipore, goat polyclonal, #AB144P, lot: 2929343; RRID: AB_2079751	1:1000
FoxP2	Recombinant human FOXP2 isoform 1 Ala640‐Glu715	R&D Systems, sheep polyclonal #AF5647; RRID: AB_2107133	1:3000
Green fluorescent protein	Full length green fluorescent protein from the jellyfish *Aequorea victoria*	ThermoFischer Scientific, Chicken, #A10262, Lot: 1972783, RRID: AB_2534023	1:3000
Lmx1b	Full‐length LIM homeobox transcription factor 1 beta protein from mouse	C. Birchmeier, Max Delbruck Center for Molecular Medicine, Berlin; guinea pig polyclonal; RRID: AB_2314752	1:8000
Neuropeptide S	Synthetic NPS peptide	Abcam, rabbit polyclonal, #ab18252, lot: GR198504‐1, RRID: AB_776718	1:3000
Parvalbumin	Frog muscle parvalbumin	Sigma‐Aldrich, Mouse, Cat# SAB4200545, lot: 066M4815V, RRID: AB_2857970	1:1000

For replication experiments labeling *Nps* mRNA in brain regions containing unexpected *Nps* Cre‐reporter expression, we used the Multiplex Fluorescent Reagent Kit v2 (ref# 323100; Advanced Cell Diagnostics). Tissues were removed from cryoprotectant, mounted, and allowed to dry for 1 h and stored at −80°C. The slides were allowed to equilibrate to room temperature, washed with PBS for 5 min, baked for 30 min at 60°C, and then postfixed in 10% neutral buffered formalin (NBF) for 15 min at 40°C. Slides were then dehydrated in a series of 50% ethanol, 70% ethanol, and 100% ethanol solutions, for 5 min each. After repeating the 100% ethanol step, slides were allowed to dry at room temperature. Sections were incubated in hydrogen peroxide (#322380; Advanced Cell Diagnostics) for 10 min at room temperature, then washed with Ultrapure water in two 2‐min washes. We next outlined sections to form a hydrophobic barrier using an ImmEdge pen (Vector Laboratories). The slides were then covered with Protease III, incubated for 30 min at 40°C, then washed for 2 min, twice using Ultrapure water. The sections were then incubated in RNAscope probes (Table 2) for 2 h at 40°C. After that, AMPs 1−3 were added, in series, for 15−30 min each, at 40°C, with two 2‐min rinses in 1× RNAscope Wash Buffer (#320058; diluted 1:50 in ddH20) between each step. Sections were covered by channel‐specific HRP for 15 min at 40°C, then 150–200 μl Opal dye (cat#FP1496001KT; Akoya Biosciences) for 30 min at 40°C, then HRP blocker for 15 min at 40°C, with two 2‐min RNAscope WashBuffer rinses between each step. Overnight immunolabeling for L10GFP was then performed as described above.

### 2.5 Immunofluorescence

For immunofluorescence labeling, we removed the tissue sections from cryoprotectant and rinsed them in PBS before loading them into a primary antibody solution. Primary antisera (Table 3) were added to a PBS solution with 0.25% Triton X‐100 (BP151‐500, Fisher), 2% normal donkey serum (NDS, 017‐000‐121, Jackson ImmunoResearch), and 0.05% sodium azide (14314, Alfa Aesar) as a preservative (PBT‐NDS‐azide). We incubated these sections overnight at room temperature on a tissue shaker. The following morning, the sections were washed 3× in PBS and incubated for 2 h at room temperature in PBT‐NDS‐azide solution containing species‐specific donkey secondary antibodies conjugated to Cy3, Cy5, or Alexa Fluor 488, or biotin (diluted 1:500−1000; Jackson ImmunoResearch) in PBT‐NDS‐azide. If a biotinylated secondary antibody was used, tissue sections were then washed three times and incubated for 2 h in streptavidin‐Cy5 (#SA1011, Invitrogen) diluted 1:1000 in PBT‐NDS‐azide. The sections were then washed three times in PBS, mounted on glass slides (#2575‐PLUS; Brain Research Laboratories), dried, and then coverslipped using Vectashield (Vector Laboratories). Slides were stored at 4°C until imaging.

### 2.6 Imaging, analysis, and figures

All slides were scanned using an Olympus VS120 microscope. We began by first acquiring a 2× overview scan then using an 10× objective to scan all tissue sections. We then acquired 20×, and in some cases 40× z‐stacks encompassing all regions of interest for this study. For each slide, this produced a Virtual Slide Image (VSI) file containing a 10× whole‐slide layer, plus separate layers with 20× and/or 40× extended‐focus images in regions of interest.

We used cellSens (Olympus) to count cells that contained *Nps* mRNA labeling in four cases with high‐quality labeling in rostral and caudal sections to count and measure *Nps* neurons. We used Abercrombie correction as an approximate way to adjust for double‐counting at the tissue edges (Guillery, 2002) and then multiplied the corrected cell counts (from our 1‐in‐3 tissue series) by 3 for a rough estimate of the number of *Nps*‐expressing neurons in a mouse brainstem. Cell counts and measurements are expressed as mean ± standard deviation. We used QuPath (Bankhead et al., 2017) to count cells expressing *Nps* mRNA, *Nmb* mRNA, and/or L10GFP in sections from Nps‐2A‐Cre;R26‐lsl‐L10GFP mice. We counted every cell that contained in‐focus labeling of at least 10 *Nps* mRNA puncta, and we did not count any lone, diffuse, or out‐of‐focus puncta. To identify cells with L10GFP expression across all z‐stacks, we counted every cell that had cytoplasmic L10GFP and a nuclear void in focus. All counts were reviewed by a senior neuroanatomist (J.C.G.) in conjunction with R.Z. and D.H. to reach consensus.

After reviewing data in OlyVIA (Olympus), we used cellSens or QuPath to crop full‐resolution images and Adobe Photoshop to adjust brightness and contrast. We used Adobe Illustrator to make drawings, plot cells for figures, arrange images, and add lettering for figure layouts. Scale bars were traced in Illustrator atop calibrated lines from cellSens or QuPath to produce clean white or black lines in each figure.

### 2.7 Nomenclature

For rat and mouse genes, we used MGI nomenclature. For rat and mouse proteins and Cre‐reporters, we used common abbreviations from the published literature. For neuroanatomical structures and cell populations, where possible, we used and referred to nomenclature defined in peer‐reviewed neuroanatomical literature. In some instances, we used or referred to nomenclature derived from rodent brain atlases (Dong, 2008; Paxinos & Franklin, 2013; Paxinos & Watson, 2007; Swanson, 1992).

## 3 RESULTS

### 3.1 *Nps*‐expressing neurons in the PB region

Overall, very few cells contained *Nps* mRNA. We counted an average of 119 ± 41 *Nps*‐expressing cells (bilateral counts from *n* = 4 mice) in a 1‐in‐3 series of brainstem sections spanning the central midbrain and rostral medulla. Multiplying by 3 and Abercrombie‐correcting each count (Guillery, 2002) suggested that the mouse brainstem contains approximately 300 *Nps*‐expressing cells (278 ± 63).

*Nps*‐expressing cells had a distinctly neuronal appearance. They were medium‐sized, slightly oblong or round, with a long‐axis diameter of 17.1 ± 3.5 μm (range 9.5−27.6 μm; *n* = 160). Their neuronal morphology was also evident from abundant *Nps* mRNA content, which in many spilled out from the soma into one or two proximal dendrites.

**FIGURE 2:**
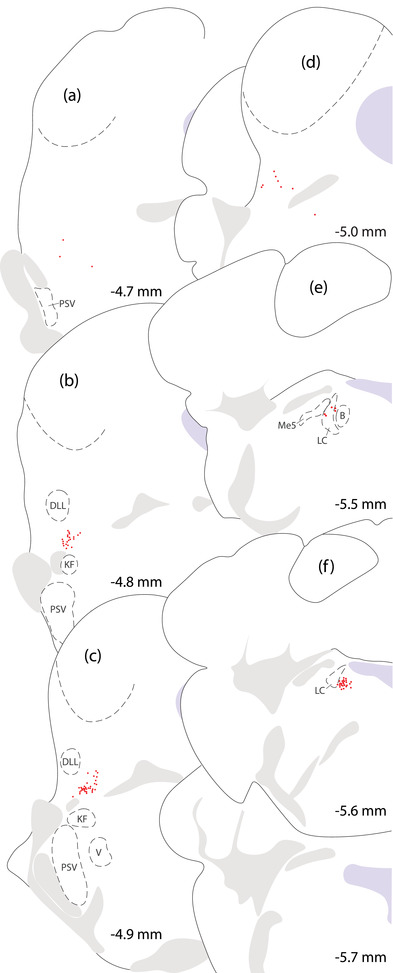
Distribution of *Nps*‐expressing neurons in the mouse parabrachial (PB) region. Neurons containing *Nps* mRNA (red dots) form a rostral cluster in the lateral parabrachial nucleus, dorsal to the Kölliker‐Fuse nucleus (KF) and ventral to the dorsal nucleus of the lateral lemniscus (DLL, b–c). Few Nps‐expressing neurons extend further rostrally (a). Caudal to this cluster (d), sparse *Nps*‐expressing neurons scatter through the PB. Further caudally (f), another dense cluster concentrates medial to the locus coeruleus (LC). *Nps*‐expressing neurons at the rostral edge of this cluster intersperse among the LC, mesencephalic trigeminal nucleus, and Barrington's nucleus (e). Approximate rostrocaudal levels (in mm, relative to bregma) are shown at bottom‐right for each brain level

*Nps*‐expressing neurons concentrated in two clusters straddling the midbrain‐hindbrain junction (Figure 2). Their location, number, and distribution were similar on the left and right. The rostral cluster concentrated in the midbrain, along the lateral edge of the rostral PB. The caudal cluster concentrated in the hindbrain, just medial to the caudal LC. There were no apparent differences in the size or appearance of *Nps* neurons between the rostral and caudal clusters. In tissue sections extending from midbrain levels containing the inferior colliculus back through hindbrain levels containing the facial nucleus, we did not find *Nps*‐expressing cells outside the PB region. In several cases, we labeled the ubiquitously expressed transcript *Ubc* in combination with *Nps*. The cytoarchitectural background revealed by *Ubc* mRNA helped clarify the neuroanatomical locations of *Nps*‐expressing neurons.

**FIGURE 3:**
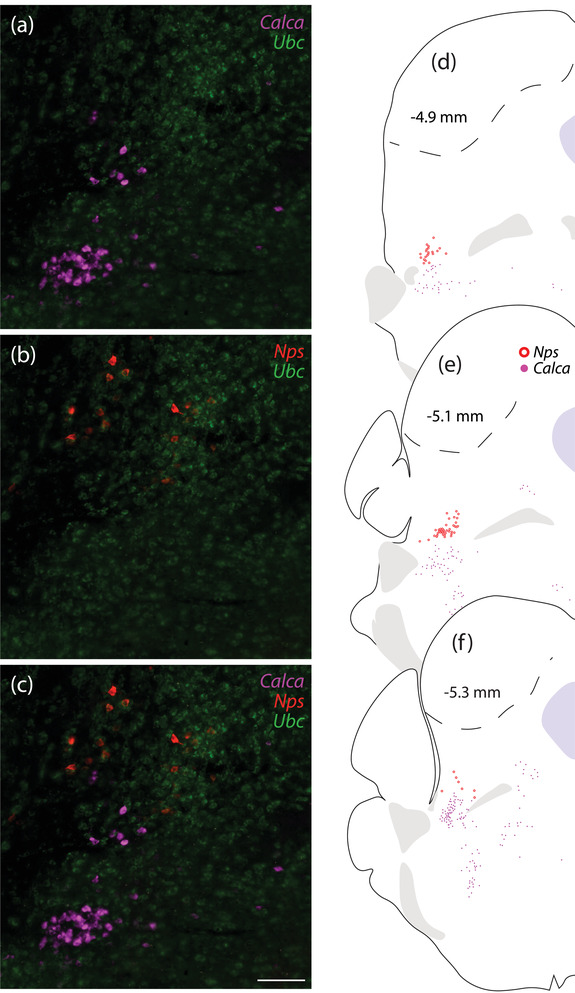
*Nps‐*expressing neurons in the rostral PB are dorsal to *Calca‐*expressing neurons. (a–c) Neurons that contain *Nps* mRNA (red) are mutually exclusive with and dorsal to neurons that contain *Calca* mRNA (magenta). *Ubc* mRNA (green) is shown for neuroanatomical background. (d–f) Rostral‐to‐caudal plot of *Nps* and *Calca* mRNA labeling. Scale bar in (c; applies to a and b) is 100 μm

The rostral cluster of *Nps*‐expressing neurons concentrated along the lateral edge of the far‐rostral PB (Figure 2b and c). Few, scattered neurons extended caudally through the lateral PB. We did not find any *Nps*‐expressing neurons in the external lateral PB subnucleus, which contained a dense collection of *Calca*‐expressing neurons. *Nps*‐expressing neurons were always dorsal to the rostral collection of *Calca*‐expressing neurons (Figure 3), and in contrast to previous reports (Adori et al., 2016; Clark et al., 2011; Liu et al., 2011; Xu et al., 2004; 2007), we did not find any *Nps*‐expressing neurons in the Kölliker‐Fuse nucleus or principal sensory trigeminal nucleus.

**FIGURE 4:**
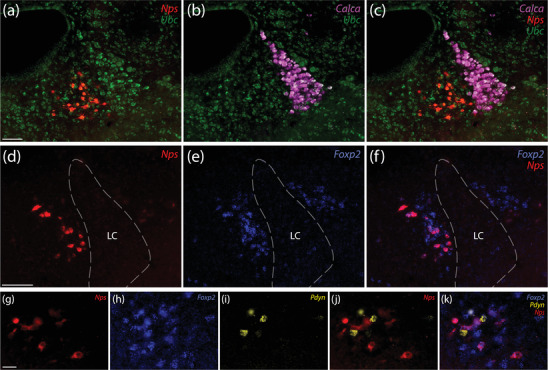
Near the LC, *Nps* colocalizes with *Foxp2* and is mutually exclusive with both *Calca* and *Pdyn*. (a–c) Neurons that contain *Nps* mRNA (red) are mutually exclusive with and medial to neurons in the LC, which contain *Calca* mRNA (magenta). *Ubc* mRNA (green) is shown for neuroanatomical background. (d–f) A subset of *Foxp2*‐expressing neurons (blue) also express *Nps* (red) medial to the LC. (g–k) This *Nps*‐expressing subset is mutually exclusive with *Pdyn* mRNA (yellow). Scale bars are 100 μm (a–c), 50 μm (d–f), 20 μm (g–k)

The caudal cluster of *Nps*‐expressing neurons concentrated beneath the floor of the fourth ventricle, in the pontine central gray matter (Figure 2f). Hindbrain tissue sections containing this cluster typically also contained the genu or post‐genu fascicles of the facial nerve, as well as the caudal LC. We did not find *Nps* mRNA in any LC neurons, which uniformly expressed *Calca* (Figure 4c). A handful of *Nps*‐expressing neurons extended rostrally and intermingled with neurons in the LC and Barrington's nucleus (Figure 2e). We did not find any colocalization between these scattered *Nps*‐expressing neurons and the distinctively larger neurons in the LC, Barrington's nucleus, or mesencephalic trigeminal nucleus.

### 3.2 *Foxp2* colocalization

**FIGURE 5:**
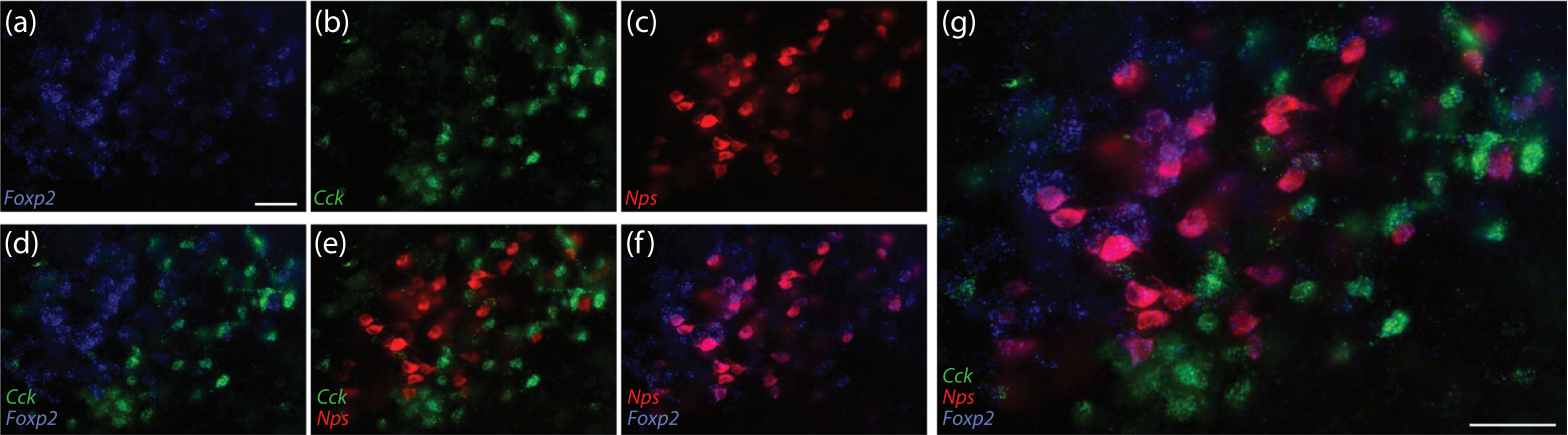
In the lateral PB, *Nps* colocalizes with *Foxp2* and is mutually exclusive with *Cck*. (a–c) Rostral distributions of neurons containing *Foxp2* (blue), *Cck* (green), or *Nps* (red) mRNA. (d) Some neurons here express both *Foxp2* and *Cck*. (e) *Nps*‐expressing neurons are mutually exclusive with *Cck*‐expressing neurons. (f) All *Nps‐*expressing neurons also express *Foxp2*. (g) Merged image. Scales bars in (a; applies to b–f) and in (g) are 50 μm

*Nps*‐expressing neurons overlap PB subregions where many glutamatergic neurons express the transcription factor *Foxp2* (Karthik et al., 2022). Using in situ hybridization to determine the relationship between this population and *Nps*‐expressing neurons, we found uniform colocalization between *Nps* and *Foxp2* (Figures 4 and 5). Every *Nps*‐expressing neuron also expressed *Foxp2*. Conversely, *Nps*‐expressing neurons were a small subset of the much larger population of *Foxp2*‐expressing neurons in this region.

### 3.3 *Nps* and other neuropeptidergic *Foxp2* subpopulations

Among *Foxp2*‐expressing neurons located caudally, the *Nps*‐expressing subset concentrated medial to the LC. We next examined their relationship to the “pre‐LC” population of neurons coexpressing *Foxp2* and *Pdyn*, which distribute through the LC, mesencephalic trigeminal nucleus, and medial PB (Gasparini et al., 2021). Labeling *Nps* and *Pdyn* in combination with *Foxp2* mRNA (Figure 4g–k) confirmed the uniform colocalization between *Foxp2* and *Nps* and between *Foxp2* and *Pdyn*. However, no neurons expressed both *Nps* and *Pdyn*, indicating that these two neuropeptide genes identify mutually exclusive subsets of *Foxp2*‐expressing neurons surrounding the LC. In the rostral lateral PB, fewer neurons expressed *Pdyn*, but the same pattern held; *Nps* and *Pdyn* each colocalized with *Foxp2*, but not with each other (not shown).

The rostral lateral PB contains a large population of neurons that express the neuropeptide *Cck*, many of which also express *Foxp2* (Garfield et al., 2014; Grady et al., 2020). To examine the relationship between *Nps* and *Cck*, we labeled mRNA for both genes in combination with *Foxp2*. Again, we found uniform colocalization between *Nps* and *Foxp2*. We also found *Foxp2* expression in many *Cck*‐expressing neurons, but *Nps* and *Cck* were mutually exclusive (Figure 5). Caudally, near the LC, fewer neurons expressed *Cck* and were again mutually exclusive with *Nps* (not shown).

### 3.4 *Nps*‐expressing PB neurons are *Atoh1*‐derived

Many *Foxp2*‐expressing neurons in the PB region derive from embryonic precursors that express the transcription factor *Atoh1* (Gray, 2008; Karthik et al., 2022). However, this region also contains *Foxp2*‐expressing inhibitory neurons, which derive from *Ptf1a*‐expressing precursors (Geerling et al., 2017; Gray, 2008; Karthik et al., 2022), and a rostral subset of *Foxp2*‐expressing neurons derives from *Lmx1a/b*‐expressing embryonic precursors (Karthik et al., 2022). To determine the origin of *Nps*‐expressing neurons, we used Cre fate‐mapping for *Atoh1*.

**FIGURE 6:**
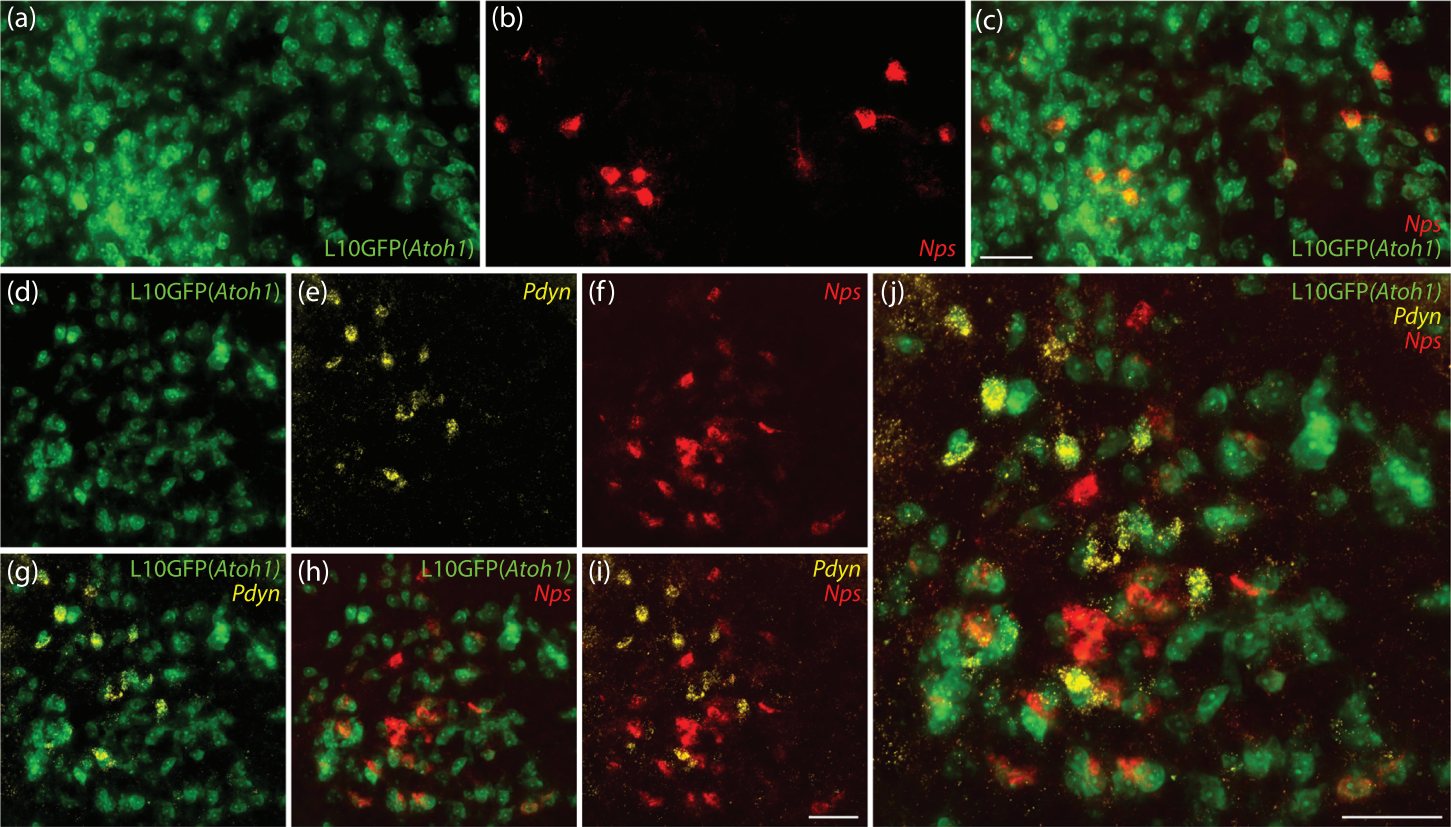
*Nps*‐expressing neurons derive from cells that express the transcription factor *Atoh1*. Cre‐dependent expression of L10GFP (green) in Atoh1‐Cre;R26‐lsl‐L10GFP mice revealed *Atoh1*‐derived neurons. *Nps* mRNA (red) colocalized with L10GFP rostrally in the lateral PB (a–c) and caudally near the LC (d–j). *Pdyn* mRNA (yellow) also colocalized with L10GFP, but *Pdyn* and *Nps* were mutually exclusive. Scale bars in (c; also applies to a and b), (i; also applies to d–h), and (j) are 50 μm

In brainstem tissue from Atoh1‐Cre;R26‐lsl‐L10GFP Cre‐reporter mice, we used in situ hybridization to label *Nps* mRNA. Virtually every *Nps*‐expressing neuron in the rostral cluster (Figure 6a–c), caudal cluster (Figure 6d–j), and scattered neurons in‐between expressed L10GFP, indicating previous expression of *Atoh1*. We also labeled *Pdyn* mRNA in these cases, which was again mutually exclusive with *Nps*. These two neuropeptides identified separate, partly intermingled subpopulations, each a subset of the larger population of *Atoh1*‐derived (L10GFP‐expressing) neurons in the PB region. We observed sparse labeling for *Pdyn* mRNA (but never *Nps*) in a small number of the distinctively larger LC neurons, none of which expressed L10GFP, complementing our previous finding that a small number of LC neurons expressed a Cre‐conditional construct in Pdyn‐IRES‐Cre mice (Grady et al., 2020).

**FIGURE 7:**
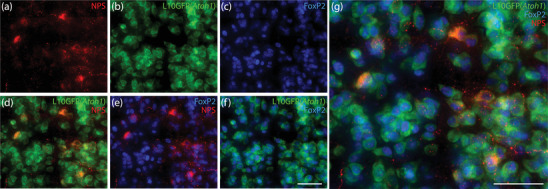
NPS‐immunoreactive neurons derive from cells that express the transcription factor *Atoh1*. Cre‐dependent expression of L10GFP (green) in Atoh1‐Cre;R26‐lsl‐L10GFP mice revealed *Atoh1*‐derived neurons. Neurons containing NPS immunofluorescence (red, a) expressed L10GFP (b) and contained FoxP2 nuclear immunofluorescence (blue, c) in the rostral lateral PB. (d–g) Merged images. Scale bars in (f; also applies to a–e) and (g) are 50 μm

**FIGURE 8:**
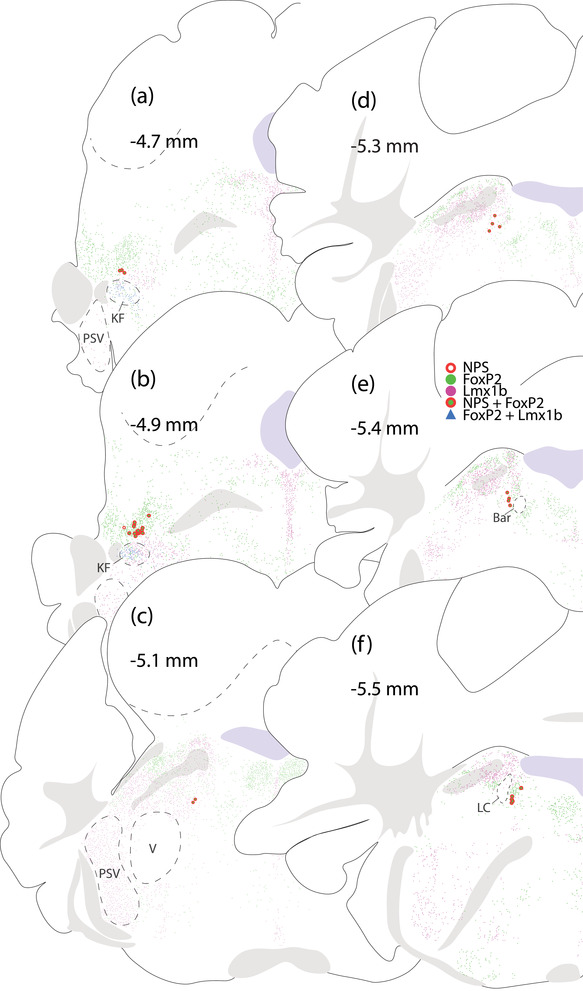
Distribution of NPS‐, FoxP2‐, and Lmx1b‐immunoreactive neurons in the PB region. (a and b) NPS‐immunoreactive neurons appear at far‐rostral levels of the PB, dorsal to KF neurons that contain both Foxp2 and Lmx1b. (c–f) Caudal to this cluster, additional NPS‐immunoreactive neurons extend medially, through the LC. Approximate rostrocaudal levels (in mm, relative to bregma) are shown at top left for each brain level. Additional PSV, principal sensory trigeminal nucleus; V, motor trigeminal nucleus; Bar, Barrington's nucleus

*Atoh1*‐derived neurons are mutually exclusive with an intermingled macropopulation of PB neurons that contain the transcription factor Lmx1b (Karthik et al., 2022). To test whether any NPS neurons contain Lmx1b, we used immunofluorescence labeling. NPS immunolabeling identified a small number of neurons with the same neuroanatomical distribution as *Nps* mRNA. As reported by others (Adori et al., 2016), the intensity of NPS immunoreactivity varied widely case‐to‐case, making it difficult to identify neuronal somata in some cases. Across four mice with the strongest labeling, we counted between 9 and 71 NPS‐immunoreactive neurons bilaterally (43 ± 29). As with *Nps* mRNA, NPS‐immunoreactive neurons uniformly expressed the L10GFP Cre‐reporter for *Atoh1* (Figure 7). Also, they contained nuclear immunoreactivity for FoxP2, not Lmx1b (Figure 8). Labeling Lmx1b and FoxP2 also confirmed that all rostral NPS‐immunoreactive neurons are dorsal to the Kölliker‐Fuse nucleus (Figure 8a and b).

### 3.5 *Nps* Cre‐reporter in the PB region

**FIGURE 9:**
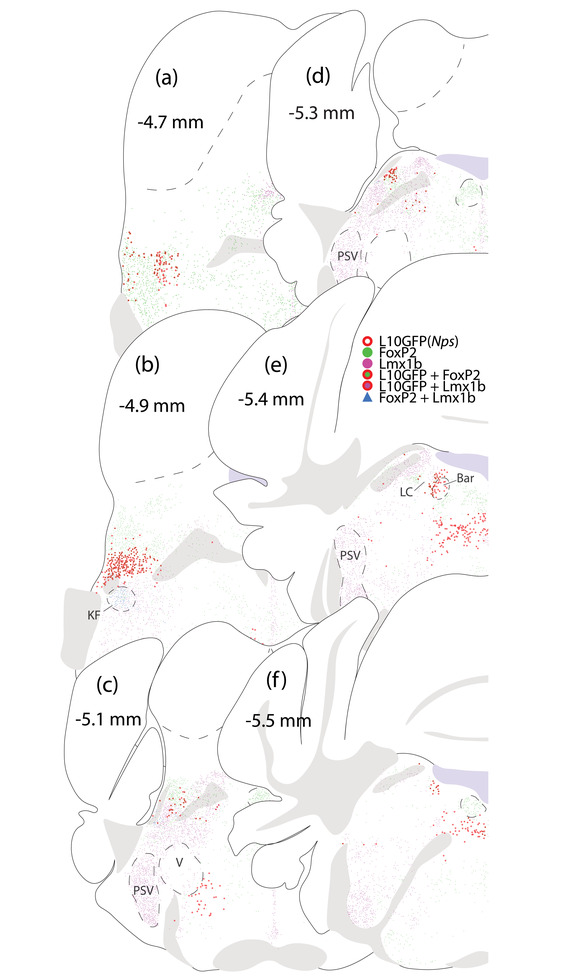
Distribution of L10GFP‐expressing, Lmx1b‐, and Foxp2‐immunoreactive neurons in the PB region of an Nps‐2A‐Cre;R26‐lsl‐L10GFP mouse. (a–d) L10GFP‐expressing neurons extended from the edge of the lateral lemniscus through the lateral PB. (e and f) Caudally, L10GFP‐expressing neurons were medial to the LC, including a large population in the nucleus incertus region

We used Nps‐2A‐Cre;R26‐lsl‐L10GFP Cre‐reporter mice to examine the distribution of cells that have expressed *Nps*. The most prominent cluster of L10GFP‐expressing neurons was in the rostral lateral PB (Figures 9a–d). Additional L10GFP‐expressing neurons extended caudally through the lateral PB, and a separate cluster formed medial to the LC (Figure 9e).

**FIGURE 10:**
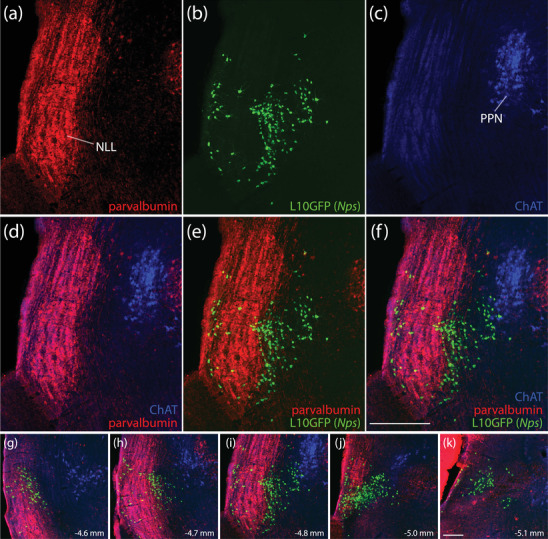
Rostral neurons expressing L10GFP (*Nps* Cre‐reporter) wrap around the nucleus of the lateral lemniscus (NLL) and extend into the lateral PB. L10GFP‐expressing neurons (b, green) are distinct from NLL neurons, which contain parvalbumin immunoreactivity (a, red). They are also distinct from pedunculopontine tegmental nucleus (PPN) that contain choline acetyltransferase immunoreactivity (ChAT; c, blue). (d–f) Merged images. *Nps* Cre‐reporter neurons appear rostrally, atop the ventral NLL (g), then wrap around the lateral lemniscus (h and i) and concentrate in the rostral lateral PB (j and k). Scale bars in (f; also applies to a–e) and (k; also applies to g–j) are 200 μm

**FIGURE 11:**
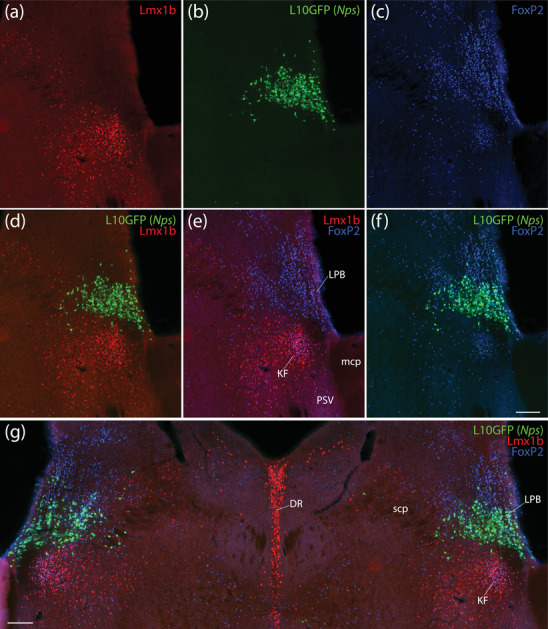
In the rostral lateral PB, neurons expressing the L10GFP Cre‐reporter for *Nps* contain FoxP2 but not Lmx1b. In this region, immunoreactivity for Lmx1b (red, a) and FoxP2 (blue, c) define mutually exclusive populations of neurons except in the KF, which contains neurons coexpressing both transcription factors (e). L10GFP‐expressing neurons (green, b) are dorsal to Lmx1b‐immunolabeled neurons (d) and contain FoxP2 immunolabeling (f). These L10GFP‐expressing neurons are dorsal to the KF in the lateral PB (g). Scale bars in (f; also applies to a–e) and (g) are 500 μm. Additional mcp, middle cerebellar peduncle; DR, dorsal raphe nucleus

Rostral L10GFP‐expressing neurons in this region were distinct from cholinergic neurons in the pedunculopontine tegmental nucleus, which contain choline acetyltransferase (ChAT, Figure 10f). These neurons wrapped around the nucleus of the lateral lemniscus, whose neurons contain parvalbumin (Figure 10e). The rostral‐most L10GFP‐expressing neurons bordering the nucleus of the lateral lemniscus contained little or no *Nps* mRNA. In contrast, the dense cluster of L10GFP‐expressing neurons in the rostral lateral PB resembled the distributions of *Nps* mRNA and NPS immunolabeling described above, and we found *Nps* mRNA in 37% of these neurons (373 of 1009 rostral PB neurons across *n* = 3 cases). This dense cluster was dorsal to Kölliker‐Fuse neurons coexpressing Lmx1b and FoxP2 (Figures 9 and 11) and rostral to lateral PB neurons expressing Lmx1b (Figure 9b–d). Virtually all L10GFP‐expressing neurons contained FoxP2 (Figure 11), and very few (<5 per case) contained Lmx1b. Across the three cases analyzed, only two neurons in one case expressed *Nps* without L10GFP.

**FIGURE 12:**
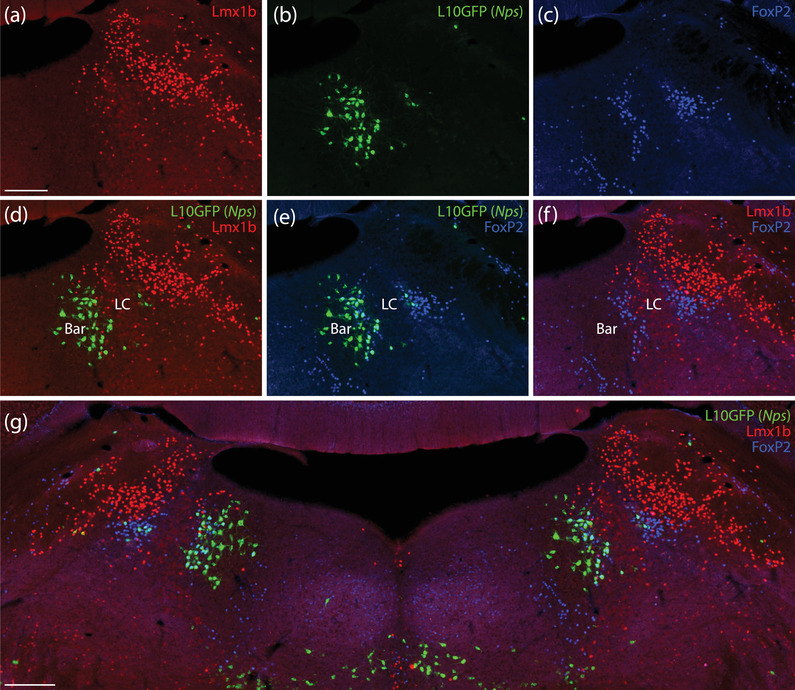
The caudal cluster of *Nps* Cre‐reporter neurons is medial to the LC, with some neurons extending into Barrington's nucleus (Bar). Most L10GFP‐expressing neurons (green, b) in this region contained FoxP2 (blue, c) and not Lmx1b (red, a). FoxP2 and Lmx1b identified mutually exclusive neuronal populations, with FoxP2‐immunoreactive neurons encircling Bar and light Lmx1b immunoreactivity identifying neurons in the LC (f). (d–g) Merged images. Bar contained several L10GFP‐expressing neurons that lacked FoxP2. Scale bars in (a; also applies to b–f) and (g) are 200 μm

Caudal to this prominent cluster, fewer L10GFP‐expressing neurons extended through the lateral PB. Their distribution avoided the Lmx1b macropopulation and formed a notable cluster among other FoxP2‐containing neurons in the dorsal lateral PB (Figure 9d). We found few L10GFP‐expressing neurons in the medial PB. As with *Nps* mRNA labeling and NPS immunolabeling, we found another prominent cluster of L10GFP‐expressing neurons medial to the locus coeruleus. Most neurons in this cluster contained FoxP2, but a small number of larger neurons within Barrington's nucleus did not (Figure 12e).

### 3.6 *Nps* Crereporter in the nucleus incertus

**FIGURE 13:**
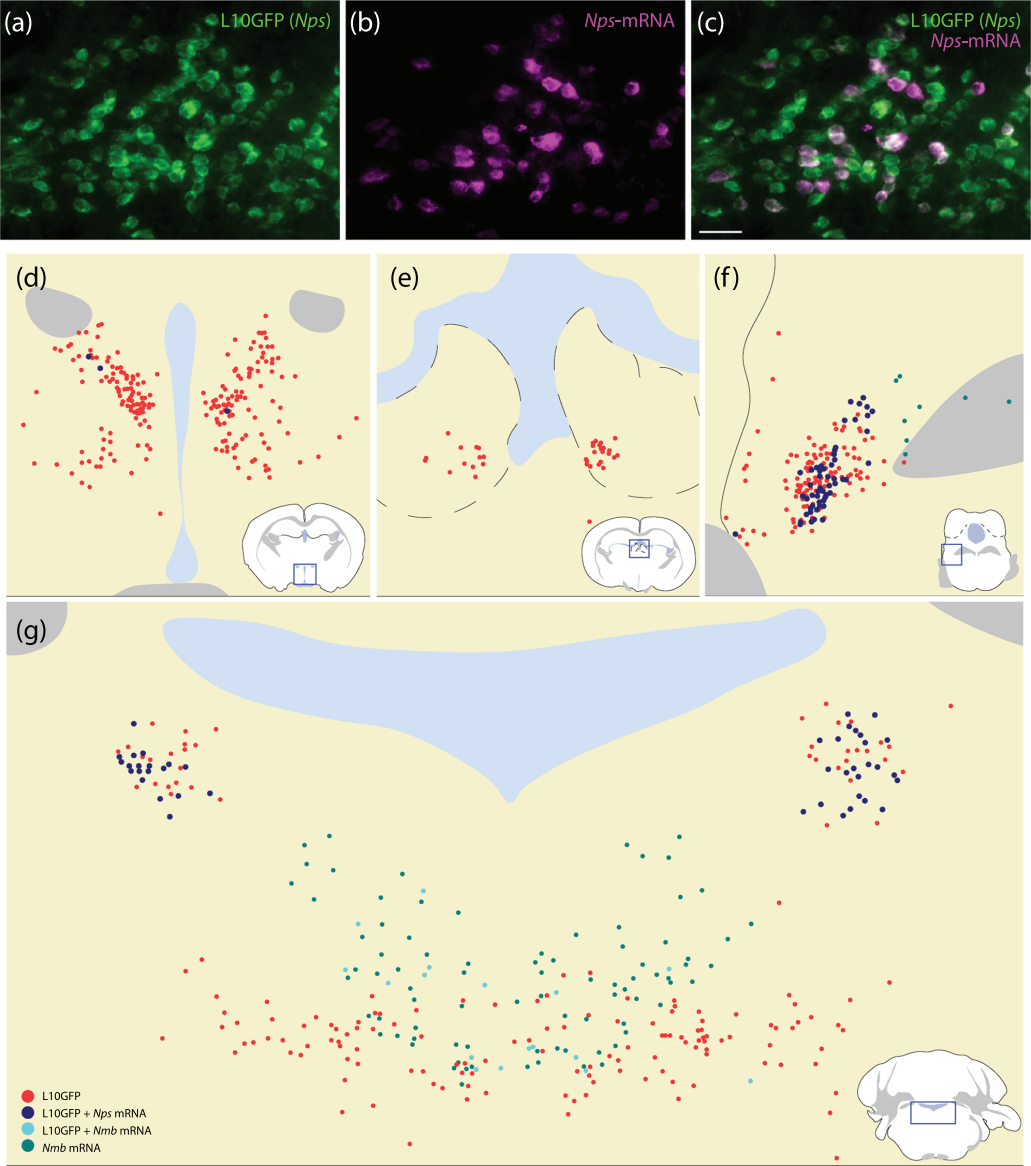
In situ hybridization of *Nps* and *Nmb* in Nps‐2A‐Cre;R26‐LSL‐L10GFP mice. Many L10GFP‐expressing neurons (green, a) also contain *Nps* mRNA (magenta, b) in the lateral PB (c, f). The anterior hypothalamus (d) and lateral habenular nucleus (e) contained additional L10GFP‐expressing neurons. Sparse L10GFP‐expressing neurons in these regions contained *Nps* mRNA (blue). More L10GFP‐expressing neurons contained *Nps* mRNA in the PB region (f and g). In the nucleus incertus, *Nmb* mRNA labeling extended dorsally while L10GFP expression extended laterally, with some neurons expressing both L10GFP and *Nmb* (g). Scale bar in (c) is 50 μm and applies to (a and b)

We found a large population of L10GFP‐expressing neurons spanning the midline of the pontine central gray matter, between the left and right cranial nerve VII genu (Figure 9e and f). Their distribution included a region referred to as “nucleus incertus” (Dong, 2008) or “central gray alpha/beta/gamma” (Paxinos & Franklin, 2013). Within this region, L10GFP‐expressing neurons intermingled with neurons expressing neuromedin B (*Nmb*) (Figure 13g), and a minority of these neurons coexpressed *Nmb*. In one case, 18 L10GFP‐expressing neurons contained *Nps* mRNA and 10 of these also contained *Nmb* mRNA. In the same case, 10 neurons contained both *Nps* and *Nmb* mRNA, without L10GFP. The remaining two cases had comparable *Nmb* labeling but only 1 and 6 nucleus incertus L10GFP‐expressing neurons contained *Nps* mRNA. Generally, the *Nmb* distribution tended dorsally, while the L10GFP distribution tended laterally (Figure 13g), including a few, scattered neurons in the reticular formation.

### 3.7 *Nps* Cre‐reporter in the habenula and hypothalamus

**FIGURE 14:**
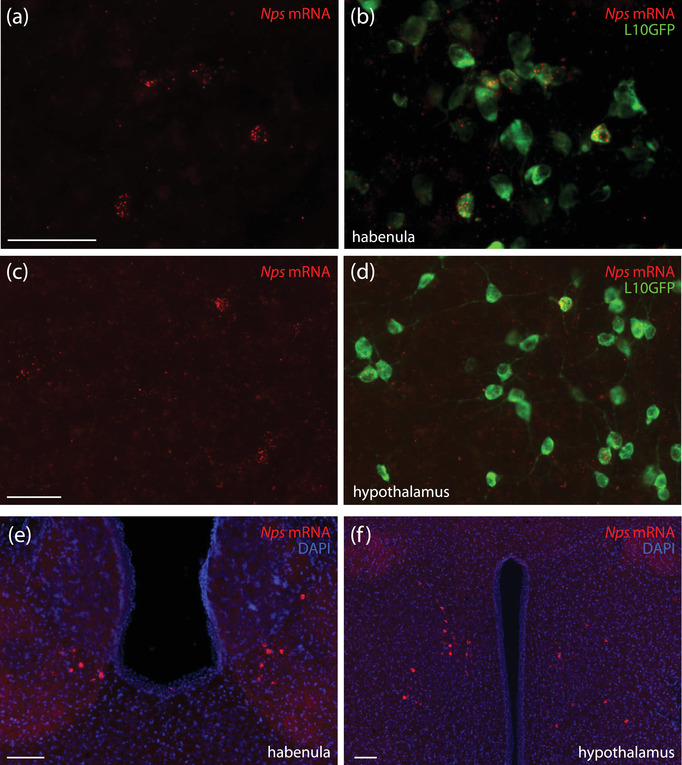
*Nps* mRNA labeling in the habenula and anterior hypothalamus. In the habenula (a and b) and hypothalamus (c and d) of Nps‐2A‐Cre;R26‐LSL‐L10GFP mice, some L10GFP‐expressing neurons contained *Nps* mRNA (red). We found a similar pattern of *Nps* mRNA labeling in mice without Nps‐2A‐Cre (e and f). Scale bars in (a) and (c) are 50 μm and apply to adjacent panels. Scale bars in (e) and (f) are 200 μm

In the forebrain, we found two additional clusters of neurons expressing the *Nps* Cre‐reporter—one in the epithalamus and the other in the anterior hypothalamus (Figure 13). The distribution of L10GFP expression in the epithalamus clustered ventrally in the lateral habenular nucleus, along its border with the medial habenular nucleus (Figure 13e). Fewer neurons extended caudally, into the pretectal region, or ventrally, into the paraventricular thalamic nucleus. In the hypothalamus, L10GFP‐expressing neurons clustered ventral to the rostral paraventricular hypothalamic nucleus. In both the habenula and hypothalamus, a minority of L10GFP‐expressing neurons contained *Nps* mRNA (Figure 14a–d). We found the same patterns of *Nps* mRNA labeling in mice without Nps‐2A‐Cre (Figure 14e and f). Just rostral to the hypothalamic cluster, we found a few, scattered neurons in the lateral preoptic area, near the bed nucleus of the stria terminalis. Immediately lateral to the hypothalamus, we found a small cluster of neurons in the medial amygdala.

### 3.8 Additional *Nps* Cre‐reporter expression

Due to these unexpected sites of Cre‐reporter expression, we analyzed the full brain in a series of Nps‐2A‐Cre;R26‐lsl‐L10GFP mice (*n* = 10). In a 1‐in‐3 series of tissue sections spanning the mouse brain, we counted 1770 ± 425 L10GFP‐expressing neurons. After Abercrombie correction and multiplication by 3, this represents approximately 3789 ± 900 neurons in the mouse brain with a history of *Nps* expression. L10GFP‐containing neurons had a similar size compared to *Nps* mRNA containing neurons, with a long‐axis diameter of 16.0 ± 1.7 μm (range 13.8−19.7 μm; *n* = 300). There were no differences in the neuroanatomical distribution and appearance of L10GFP‐containing neurons among cases, and the overall number did not differ between male (3869 ± 1015; *n* = 6) and female (3669 ± 827; *n* = 4) Nps‐2A‐Cre;R26‐lsl‐L10GFP mice (*p* = .75 by two‐tailed *t*‐test).

These include 2927 ± 905 (77.3%) in the brainstem, primarily in the PB, but also in the nucleus incertus and rare, scattered neurons in the rostral periaqueductal gray matter. The remaining L10GFP‐expressing neurons clustered in the hypothalamus (382 ± 154; 10.1%), thalamus (414 ± 97; 10.9%), and medial amygdala (65 ± 35; 1.7%), as described above. We did not find any L10GFP‐expressing neurons in the cerebral cortex, except for a lone hippocampal neuron in two cases. We did not find any L10GFP‐expressing neurons in the basal ganglia, basal forebrain, or septal nuclei. We did not find any L10GFP‐expressing neurons in the cerebellum. Finally, we did not find any L10GFP‐expressing neurons in the hindbrain caudal to the nucleus incertus, but near the spinomedullary transition, we identified L10GFP expression in a small number of epithelioid cells surrounding the central canal of the spinal cord.

## 4 DISCUSSION

NPS promotes wakefulness, and genetic polymorphisms in *NPSR1* have been linked to several human diseases, but the homeostatic context and ethological significance of these effects remain uncertain. It is unknown precisely when or why NPS is released, so it is necessary to investigate *Nps*‐expressing neurons. Our results clarify their neuroanatomical distribution and identify them within the molecular ontology of the parabrachial region. After briefly reviewing what is known about neuronal diversity in the PB, we will discuss *Nps*‐expressing neurons within this framework and what our findings predict about their connectivity and function. Additionally, we will discuss the unexpected finding of neurons with a history of *Nps* expression in novel neuroanatomical locations.

### 4.1 Developmental‐genetic framework of the PB region

The brainstem tegmentum at the midbrain‐hindbrain junction contains many, diverse neurons. The larger, more distinctive neurons (locus coeruleus, mesencephalic trigeminal nucleus, laterodorsal tegmental nucleus, and Barrington's nucleus) stand out from an expansive background of smaller neurons in the PB and pontine central gray matter, which have received less attention.

The neuroanatomical boundaries subdividing these regions in current brain atlases derive from efforts in rats to organize this region using cytoarchitecture (Fulwiler & Saper, 1984; Paxinos & Watson, 2007; Swanson, 1992). Subsequent patterns of connectivity, gene and protein expression, Fos activation, and Cre fate‐mapping blurred these boundaries and identified neuronal populations that do not fit this framework (Gasparini et al., 2021; Karthik et al., 2022). Delineations in rats do not always translate cleanly to mice (Biag et al., 2012; Gasparini et al., 2019; 2021; Karthik et al., 2022) and may be even less helpful for translating findings from rodents to humans. Importantly, it is not possible to use position or cytoarchitecture in this region to draw borders between most neuronal populations because subpopulations with separate connections and functions intermingle extensively (Gasparini et al., 2019; 2021; Geerling et al., 2016; Geerling et al., 2011; Grady et al., 2020; Huang et al., 2020; 2021; Karthik et al., 2022; Miller et al., 2012).

We recently described a molecular ontology of transcription factors and other genetic markers that define and differentiate subpopulations of neurons in and around the PB (Karthik et al., 2022). In the rostral rhombic lip of the embryonic brain, neuroepithelial cells that express the transcription factor *Atoh1* generate a wide variety of neurons, including the PB, Barrington's nucleus, and most neurons in the cerebellum (Akazawa et al., 1995; Ben‐Arie et al., 1997; Machold & Fishell, 2005; Rose et al., 2009; Wang et al., 2005). Most *Atoh1*‐derived neurons in the PB express *Foxp2* and intermingle with a separate macropopulation of glutamatergic neurons, which derives from a mutually exclusive lineage marked by the transcription factors *Lmx1a* and *Lmx1b* (Karthik et al., 2022).

The distribution of *Foxp2*‐expressing glutamatergic neurons in this region extends from rostral levels of the lateral PB back through the pre‐LC. Most of these neurons are *Atoh1*‐derived (Geerling et al., 2016; Geerling et al., 2011; Shin et al., 2011; Verstegen et al., 2017), but ventromedial and ventrolateral fringe populations contain GABAergic FoxP2 neurons that are not *Atoh1*‐derived (Geerling et al., 2017; Gray, 2008; Huang et al., 2020; Karthik et al., 2022; Verstegen et al., 2017). Identifying NPS neurons as *Atoh1*‐derived and *Foxp2*‐expressing places them within the molecular ontology of the PB region.

### 4.2 NPS neurons are a subset of *Atoh1*‐derived, *Foxp2*‐expressing PB neurons: distinctions and implications

Our results add *Nps* to a growing list of neuropeptides (including *Pdyn*, *Cck*, and *Grp*) that subdivide the *Atoh1*‐derived PB macropopulation into distinct subpopulations (Karthik et al., 2022).

In previous work, we identified a rostral and caudal pair of neuronal clusters that express *Foxp2*, receive direct input from aldosterone‐sensitive neurons, and activate in response to sodium deprivation (Gasparini et al., 2019; Geerling & Loewy, 2006, 2007; Geerling et al., 2011). The rostral cluster is in the lateral PB, and the caudal cluster is located near the LC (Gasparini et al., 2021). Much like these sodium‐deprivation‐activated neurons, NPS neurons form distinctive rostral and caudal clusters. However, their molecular identity is distinct. In contrast to NPS neurons, sodium‐deprivation‐activated neurons express *Pdyn* (Gasparini et al., 2021; Lee et al., 2019). Also, most of the caudal NPS cluster is medial to the locus coeruleus, while the distribution of “pre‐LC” sodium‐deprivation‐activated neurons skews laterally, into the medial PB (Gasparini et al., 2021). Based on this genetic and neuroanatomical dissection, we predict that *Nps*‐expressing neurons do not respond to sodium depletion and do not play a role in sodium appetite.

Considering that NPS reduces feeding (Smith et al., 2006) and that glutamatergic neurons near the LC reduce food and water intake (Gong et al., 2020; Li et al., 2019), it is tempting to speculate that NPS neurons relay meal‐related information from the NTS to the hypothalamus, constraining food or fluid consumption. It remains unknown whether such viscerosensory information reaches any NPS neurons or how that information interacts with wake‐promoting effects of NPS. Of note, meal timing influences circadian rhythm (Bolles & Stokes, 1965; Boulos et al., 1980; Mistlberger, 1994), and NPS neurons may serve as a bridge between meal‐related viscerosensory information and the diencephalic neurons that control sleep/wake switching and other aspects of circadian physiology.

Clearly, a more complete understanding of the NPS system will require learning what activates *Nps*‐expressing neurons. In a previous study, restraint stress and forced‐swim stress activated neurons in the PB region expressing an *Nps*‐eGFP transgene (Liu et al., 2011). Beyond this, we have very little information about NPS neuron activity, and we do not know what input signals they receive. Neighboring pre‐LC neurons integrate input from the NTS, hypothalamus, bed nucleus of the stria terminalis, and cerebral cortex (Gasparini et al., 2019; 2021; Kelly & Watts, 1998; Lee et al., 2019; Li et al., 2019), but we do not yet know if NPS neurons receive input from these regions.

Regarding their output connectivity, learning that NPS neurons express *Foxp2* and derive from *Atoh1*‐expressing precursors provides a clue to the list of brain regions they are likely to target (Huang et al., 2020; 2021; Karthik et al., 2022). Anterograde and retrograde tracing in rats and mice defined the array of brain regions that do and do not receive input from *Foxp2*‐expressing neurons, relative to other glutamatergic and GABAergic populations in the PB region (Geerling et al., 2017; Huang et al., 2020; 2021). Based on this information, *Nps*‐expressing neurons in the PB region probably do not project axons to the cerebral cortex, basal forebrain, amygdala, or most regions of the hindbrain. This is noteworthy due to the prominent expression of the NPS receptor (*Npsr1*) in the cerebral cortex, basal forebrain, and amygdala (Clark et al., 2011; Xu et al., 2007). These regions may represent mismatches between NPS and its receptor (Clark et al., 2011), but our identification of *Nps*‐expressing neurons in the thalamus, hypothalamus, and medial amygdala expands the possible sources of endogenous NPS. For example, NPS release in response to stress has been detected in the amygdala using microdialysis (Ebner et al., 2011).

Based on results of conventional axonal tracing (Luppi et al., 1995; Shin et al., 2011) and Cre‐conditional tracing in Foxp2‐IRES‐Cre mice (Huang et al., 2020), we predict that NPS neurons in the PB project their axons to the paraventricular thalamic nucleus (PVT), hypothalamic subregions, and possibly the lateral septum, ventral tegmental area, or periaqueductal gray matter. These regions are known to contain NPS‐immunoreactive axons and *Npsr1*‐expressing neurons, and particularly dense axonal labeling and *Npsr1* mRNA highlight the PVT as a potentially important target (Clark et al., 2011). The expanded population of neurons with a history of *Nps* expression identified in our Cre‐reporter mice extends far more rostrally than the PB injection sites in previous Foxp2‐IRES‐Cre tracing experiments (Huang et al., 2020), so we cannot rule out the possibility of additional output targets. Planning and interpreting future experiments which evaluate possible functions of NPS neurons would benefit from a detailed, brain‐wide map of their axonal projections.

### 4.3 Distribution of *Nps*‐expressing neurons

Despite differences in neuroanatomical nomenclature, the patterns of *Nps* mRNA and NPS immunolabeling we observed in the PB region are consistent with previous reports (Adori et al., 2015; Clark et al., 2011; Xu et al., 2004). Our estimate that approximately 300 neurons contain *Nps* mRNA in this region resembles a previous report that approximately 500 neurons in the mouse brainstem expressed an *Nps*‐eGFP transgene (Liu et al., 2011).

Nps‐2A‐Cre;R26‐lsl‐L10GFP mice provided a more comprehensive understanding of neurons that have expressed *Nps*. In addition to the familiar PB distributions, we discovered unexpected populations in the nucleus incertus, lateral habenular nucleus, and anterior hypothalamus. In these novel populations, we found sparse *Nps* mRNA labeling. Our results indicate that relatively few neurons express *Nps* continuously, but more express this gene under phasic conditions that remain unknown.

Medial to the LC, Cre‐reporter expression and FoxP2 immunolabeling revealed *Nps* reporter‐expressing neurons in Barrington's nucleus. These large neurons lacked FoxP2, which identifies smaller neurons surrounding Barrington's nucleus (Verstegen et al., 2017). Neurons in Barrington's nucleus send output to the lumbosacral spinal cord to control micturition and other pelvic functions (Hou et al., 2016; Ito et al., 2020; Keller et al., 2018; Loewy et al., 1979; Verstegen et al., 2019). This nucleus contains multiple glutamatergic subpopulations (Keller et al., 2018), and it will be important to determine the role of those with a history of *Nps* expression.

Further medially, we found neurons with a history of *Nps* expression in the nucleus incertus. Neurons here send output to the interpeduncular nucleus, septum, and several other brain regions (Goto et al., 2001). Stimulating the *Nmb*‐expressing subset of nucleus incertus neurons increases locomotor speed and pupillary dilation (Lu et al., 2020). Few neurons here actively expressed *Nps* in our cases, but a sizeable minority with the *Nps* Cre‐reporter also expressed *Nmb*. It will be important to distinguish the connectivity and function of nucleus incertus neurons with a history of *Nps* expression.

Discovering *Nps* expression in the habenula was particularly surprising because a transcriptomic study of this brain region did not identify substantial *Nps* expression (Hashikawa et al., 2020). Lateral habenular neurons reduce dopamine neuron activity, which is important for negative reward prediction (Christoph et al., 1986; Jhou et al., 2009; Matsumoto & Hikosaka, 2007; Stamatakis & Stuber, 2012). It will be interesting to learn the function of habenular neurons that express *Nps*.

In the anterior hypothalamus, we identified another population of Cre‐reporter‐expressing neurons ventral to the paraventricular hypothalamic nucleus. The subparaventricular region receives input from several limbic brain regions and contains GABAergic interneurons that inhibit glutamatergic neurons in the paraventricular nucleus (Herman et al., 2002). A ventral subregion referred to as the subparaventricular zone (SPZ) relays input from the suprachiasmatic nucleus to neurons implicated in circadian function (Vujovic et al., 2015). It will be important to determine the functional role of neurons in this region that express *Nps*.

### 4.4 Limitations

The L10GFP intensity and distribution were similar across *Nps* Cre‐reporter mice and did not vary with mRNA labeling intensity. This discrepancy reflects expected differences between the dynamic transcription of endogenous *Nps* and the strong, continuous expression of L10GFP expression from the ROSA26 locus with a CAG promoter (Krashes et al., 2014). The purpose of this study was to characterize NPS neurons within the molecular framework of the PB region, and we used *Nps* knockin Cre‐reporter mice to study adult neurons with a history of *Nps* expression but did not examine peripheral tissues or embryonic *Nps* or L10GFP expression. We also did not test the functionality of the NPS(−2A) neuropeptide translated and processed from the Nps(−2A‐Cre) transcript. The variable NPS immunolabeling intensity we observed among cases could be a result of circadian factors (Ensho et al., 2017), sleep pressure (Adori et al., 2016), or stress (Liu et al., 2011).

## 5 CONCLUSIONS

The potential therapeutic importance of NPS makes it imperative that we learn more about neurons that produce it, but few studies have interrogated the connectivity and function of these neurons. Our results clarify the neuroanatomical distribution and molecular identity of NPS neurons within the diverse framework of the PB region. This information lays the groundwork for future experiments involving NPS neurons.

## AUTHOR CONTRIBUTIONS

JCG planned the project, secured funding, helped with initial histology and microscopy, and drafted the paper with RZ and DH. WJP planned and supervised the generation of knockin Nps‐2A‐Cre mice. SG bred, weaned, genotyped, and perfused Nps‐2A‐Cre;R26‐lsl‐L10GFP mice. RZ performed additional histology and microscopy. MCM and RZ performed high‐sensitivity in situ hybridization for *Nps*. DH and RZ drafted the figures. DH, RZ, and JCG edited the figures and figure legends together. RZ, DH, SG, WJP, and JCG edited and finalized the text together. All authors approved of the text and figures in this manuscript.

## FUNDING

This work was supported by an NINDS K08 award (NS099425, to JCG) and by a 2021 Accelerator Award from the Iowa Neuroscience Institute (to JCG). Knockin Nps‐2A‐Cre mice were generated at the University of Iowa Genome Editing Core Facility, which is supported, in part, by grants from the NIH and from the Roy J. and Lucille A. Carver College of Medicine.

## CONFLICT OF INTEREST

The authors declare no conflicts of interest.

### PEER REVIEW

The peer review history for this article is available at https://publons.com/publon/10.1002/cne.25400

## Data Availability

The data that support the findings of this study are available from the corresponding author upon reasonable request.
